# Estimating mortality and disability in Peru before the COVID-19 pandemic: a systematic analysis from the Global Burden of the Disease Study 2019

**DOI:** 10.3389/fpubh.2023.1189861

**Published:** 2023-06-22

**Authors:** Maria Jesus Rios-Blancas, Victoria Pando-Robles, Christian Razo, Cesar P. Carcamo, Walter Mendoza, Kevin Pacheco-Barrios, J. Jaime Miranda, Van Charles Lansingh, Takele Gezahegn Demie, Manika Saha, Osaretin Christabel Okonji, Arzu Yigit, Lucero Cahuana-Hurtado, Pamela R. Chacón-Uscamaita, Eduardo Bernabe, Carlos Culquichicon, Jesus Lorenzo Chirinos-Caceres, Rosario Cárdenas, Jacqueline Elizabeth Alcalde-Rabanal, Francisco J. Barrera, Beatriz Paulina Ayala Quintanilla, Seyed Afshin Shorofi, Nuwan Darshana Wickramasinghe, Nuno Ferreira, Louay Almidani, Vivek Kumar Gupta, Hanie Karimi, Daniel Shewaye Alayu, Catherine P. Benziger, Takeshi Fukumoto, Ebrahim Mostafavi, Elrashdy Moustafa Mohamed Redwan, Mesfin Gebrehiwot, Khaled Khatab, Ai Koyanagi, Fiorella Krapp, Seung Lee, Maryam Noori, Ibrahim Qattea, Victor Daniel Rosenthal, Joseph W. Sakshaug, Birhanu Wagaye, Iman Zare, Doris V. Ortega-Altamirano, Efrén Murillo-Zamora, Dominique Vervoort, Diego Augusto Santos Silva, Abderrahim Oulhaj, Brenda Yuliana Herrera-Serna, Rahul Mehra, Mehrdad Amir-Behghadami, Nasrin Adib, Sandra Cortés, Anh Kim Dang, Binh Thanh Nguyen, Ali H. Mokdad, Simon I. Hay, Christopher J. L. Murray, Rafael Lozano, Patricia J. García

**Affiliations:** ^1^School of Public Health of Mexico, National Institute of Public Health, Cuernavaca, Mexico; ^2^Carlos Slim Foundation, Mexico City, Mexico; ^3^Infectious Disease Research Center, National Institute of Public Health, Cuernavaca, Mexico; ^4^Institute for Health Metrics and Evaluation, University of Washington, Seattle, WA, United States; ^5^School of Public Health and Administration, Universidad Peruana Cayetano Heredia, Lima, Peru; ^6^Peru Country Office, United Nations Population Fund (UNFPA), Lima, Peru; ^7^Spaulding Rehabilitation Hospital, Harvard University, Boston, MA, United States; ^8^Universidad San Ignacio de Loyola (University of Saint Ignatius of Loyola), Lima, Peru; ^9^CRONICAS Centre of Excellence in Chronic Diseases, Universidad Peruana Cayetano Heredia (Cayetano Heredia Peruvian University), Lima, Peru; ^10^Department of Medicine, Universidad Peruana Cayetano Heredia (Cayetano Heredia Peruvian University), Lima, Peru; ^11^HelpMeSee, New York, NY, United States; ^12^Mexican Institute of Ophthalmology, Queretaro, Mexico; ^13^School of Public Health, St. Paul's Hospital Millennium Medical College, Addis Ababa, Ethiopia; ^14^Department of Humanities and Social Sciences, Deakin University, Melbourne, VIC, Australia; ^15^Department of Human-Centred Computing, Monash University, Melbourne, VIC, Australia; ^16^School of Pharmacy, University of the Western Cape, Cape Town, South Africa; ^17^Department of Health Management, Süleyman Demirel Üniversitesi (Süleyman Demirel University), Isparta, Türkiye; ^18^Emerge, Emerging Diseases and Climate Change Research Unit, School of Public Health and Administration, Universidad Peruana Cayetano Heredia, Lima, Peru; ^19^Faculty of Dentistry, Oral and Craniofacial Sciences, King’s College London, London, United Kingdom; ^20^Universidad Privada Norbert Wiener, Centro de Investigación Epidemiológica en Salud Global, Lima, Peru; ^21^Department of Health Care, Metropolitan Autonomous University, Mexico City, Mexico; ^22^Center for Health Systems Research, National Institute of Public Health, Cuernavaca, Mexico; ^23^Department of Epidemiology, Harvard University, Boston, MA, United States; ^24^The Judith Lumley Centre, La Trobe University, Melbourne, VIC, Australia; ^25^San Martin de Porres University, Lima, Peru; ^26^Medical-Surgical Nursing, Mazandaran University of Medical Sciences, Sari, Iran; ^27^College of Nursing and Health Sciences, Flinders University, Adelaide, SA, Australia; ^28^Department of Community Medicine, Rajarata University of Sri Lanka, Anuradhapura, Sri Lanka; ^29^Department of Social Sciences, University of Nicosia, Nicosia, Cyprus; ^30^Nuffield Department of Primary Care Health Sciences, University of Oxford, Oxford, United Kingdom; ^31^Doheny Image Reading and Research Lab (DIRRL) - Doheny Eye Institute, University of California, Los Angeles, Los Angeles, CA, United States; ^32^Faculty of Medicine Health and Human Sciences, Macquarie University, Sydney, NSW, Australia; ^33^School of Medicine, Tehran University of Medical Sciences, Tehran, Iran; ^34^Department of Epidemiology and Biostatistics, University of Gondar, Gondar, Ethiopia; ^35^Heart and Vascular Center, Essentia Health, Duluth, MN, United States; ^36^Department of Dermatology, Kobe University, Kobe, Japan; ^37^Department of Medicine, Stanford University, Palo Alto, CA, United States; ^38^Stanford Cardiovascular Institute, Stanford University, Palo Alto, CA, United States; ^39^Department Biological Sciences, King Abdulaziz University, Jeddah, Egypt; ^40^Department of Protein Research, Research and Academic Institution, Alexandria, Egypt; ^41^Department of Environmental Health, Wollo University, Dessie, Ethiopia; ^42^Health and Wellbeing, Sheffield Hallam University, Sheffield, United Kingdom; ^43^Biomedical Research Networking Center for Mental Health Network (CIBERSAM), Sant Boi de Llobregat, Spain; ^44^Catalan Institution for Research and Advanced Studies (ICREA), Barcelona, Spain; ^45^Instituto de Medicina Tropical Alexander von Humboldt (Alexander von Humboldt Institute for Tropical Medicine), Cayetano Heredia University, Lima, Peru; ^46^Doctoral School of Biomedical Sciences, Katholieke Universiteit Leuven, Leuven, Belgium; ^47^Department of Precision Medicine, Sungkyunkwan University, Suwon-si, Republic of Korea; ^48^Student Research Committee, Iran University of Medical Sciences, Tehran, Iran; ^49^Department of Neonatology, Case Western Reserve University, Cleveland, OH, United States; ^50^International Nosocomial Infection Control Consortium, Independent Consultant, Buenos Aires, Argentina; ^51^Institute for Employment Research, University of Warwick, Coventry, United Kingdom; ^52^Department of Statistics, Ludwig Maximilians University, Munich, Germany; ^53^Department of Public Health Nutrition, Wollo University, Dessie, Ethiopia; ^54^Infection Prevention and Control and Water, Sanitation and Hygiene Unit, Ethiopian Public Health Institute, Addis Ababa, Ethiopia; ^55^Research and Development Department, Sina Medical Biochemistry Technologies Co. Ltd., Shiraz, Iran; ^56^Health Systems Research Center, National Institute of Public Health, Cuernavaca, Mexico; ^57^Clinical Epidemiology Research Unit, Mexican Institute of Social Security, Villa de Alvarez, Mexico; ^58^Postgraduate in Medical Sciences, Universidad de Colima, Colima, Mexico; ^59^Department of Health Policy and Management, Johns Hopkins University, Baltimore, MD, United States; ^60^Department of Physical Education, Federal University of Santa Catarina, Florianópolis, Brazil; ^61^Department of Epidemiology and Population Health, Khalifa University, Abu Dhabi, United Arab Emirates; ^62^Departamento de Salud Oral (Department of Oral Health), Universidad Autónoma de Manizales (Autonomous University of Manizales), Manizales, Colombia; ^63^Food Science and Technology, Maharishi Markandeshwar (Deemed to be University), Ambala, Haryana, India; ^64^Road Traffic Injury Research Center, Iranian International Safe Community Support Center, Tabriz University of Medical Sciences, Tabriz, Iran; ^65^Department of Health Service Management, Iranian Center of Excellence in Health Management, Tabriz, Iran; ^66^Department of Veterinary Pathobiology, Shahid Bahonar University of Kerman, Kerman, Iran; ^67^Department of Public Health, Pontifical Catholic University of Chile, Santiago, Chile; ^68^Research Line in Environmental Exposures and Health Effects at Population Level, Centro de Desarrollo Urbano Sustentable (CEDEUS), Advanced Center for Chronic Diseases (ACCDIS), Santiago, Chile; ^69^Institute for Global Health Innovations, Duy Tan University, Da Nang, Vietnam; ^70^Department of Health Metrics Sciences, School of Medicine, University of Washington, Seattle, WA, United States

**Keywords:** health metrics, mortality, disability-adjusted life year (DALY), risk factors, Peru, global burden of disease

## Abstract

**Background:**

Estimating and analyzing trends and patterns of health loss are essential to promote efficient resource allocation and improve Peru’s healthcare system performance.

**Methods:**

Using estimates from the Global Burden of Disease (GBD), Injuries, and Risk Factors Study (2019), we assessed mortality and disability in Peru from 1990 to 2019. We report demographic and epidemiologic trends in terms of population, life expectancy at birth (LE), mortality, incidence, prevalence, years of life lost (YLLs), years lived with disability (YLDs), and disability-adjusted life-years (DALYs) caused by the major diseases and risk factors in Peru. Finally, we compared Peru with 16 countries in the Latin American (LA) region.

**Results:**

The Peruvian population reached 33.9 million inhabitants (49.9% women) in 2019. From 1990 to 2019, LE at birth increased from 69.2 (95% uncertainty interval 67.8–70.3) to 80.3 (77.2–83.2) years. This increase was driven by the decline in under-5 mortality (−80.7%) and mortality from infectious diseases in older age groups (+60 years old). The number of DALYs in 1990 was 9.2 million (8.5–10.1) and reached 7.5 million (6.1–9.0) in 2019. The proportion of DALYs due to non-communicable diseases (NCDs) increased from 38.2% in 1990 to 67.9% in 2019. The all-ages and age-standardized DALYs rates and YLLs rates decreased, but YLDs rates remained constant. In 2019, the leading causes of DALYs were neonatal disorders, lower respiratory infections (LRIs), ischemic heart disease, road injuries, and low back pain. The leading risk factors associated with DALYs in 2019 were undernutrition, high body mass index, high fasting plasma glucose, and air pollution. Before the COVID-19 pandemic, Peru experienced one of the highest LRIs-DALYs rates in the LA region.

**Conclusion:**

In the last three decades, Peru experienced significant improvements in LE and child survival and an increase in the burden of NCDs and associated disability. The Peruvian healthcare system must be redesigned to respond to this epidemiological transition. The new design should aim to reduce premature deaths and maintain healthy longevity, focusing on effective coverage and treatment of NCDs and reducing and managing the related disability.

## 1. Introduction

Peru is a multi-ethnic South-American nation (1) with a diverse topography characterized by three primary natural regions: the coastal area, the highlands, and the rainforest. The coastal region, constituting a desertic expanse, accommodates 58% of the country’s population. Meanwhile, the highlands, which are inhabited by approximately 28.1% of the population, predominantly comprise individuals of Quechua and Aymara ethnic descent. However, due to the limited infrastructure, specifically deficient road networks, access to essential services within this region remains constrained (2). Lastly, the rainforest or jungle region, encompassing around 13.9% of the population, encompasses an intricate network of approximately 2,703 geographically scattered native communities and ethnic groups (3). Notably, the primary urban center within this region, Iquitos, can solely be accessed *via* air transportation. Consequently, these geographical characteristics engender numerous challenges for the healthcare system, ultimately culminating in substantial disparities and inequities (1–3). Over the past 30 years, Peru has experienced significant political, economic, and social changes (4), This period witnessed a remarkable shift in the gross domestic product (GDP) annual growth rate, from −4.9% in 1990 to 2.2% in 2019. As a result, the World Bank classified Peru as a high/middle-income country. However, this classification fails to fully address the persisting inequities and the need for an ambitious development agenda to bridge the existing social gaps (5).

The healthcare system in Peru exhibits a fragmented structure (6–8). The Ministry of Health (MoH) provides most stewardship functions for healthcare services covering 61% of the total population– mainly poor and/or informal-sector workers and the unemployed and their families (9, 10). The Ministry of Labor, through the Social Security System (EsSalud, by its acronym in Spanish), provides healthcare services to the working class and their families (31%). The Peruvian Armed Forces and National Police healthcare institutions provide services to around 2% of the population, and finally, 2.7% of the population is covered by private insurance (11, 12). In 2019, the Government implemented a universal health coverage policy, ensuring the automatic affiliation of every citizen to the Integral Health System (SIS) regardless of their socioeconomic or employment status (13). However, health expenditure in Peru accounted for 5.2% of the GDP in the same year, which is lower than other countries in the region with similar income levels. For instance, Brazil allocated 8.8% of its GDP to health expenditure, and Colombia allocated 7.2% (14). Despite the changes made within the healthcare system, the availability of increased financial resources and improved managerial practices has not kept pace with this transformation. As a result, out-of-pocket health expenditures remain disproportionately high, constituting around 30 to 40% of the total health expenditures (12). In this context, it is essential to monitor the population’s health status and analyze health information and trends timely and accurate. This will provide data to inform policy, allocate resources effectively, and adapt the current healthcare system appropriately. Although some local studies have estimated the main causes of health loss in Peru (15–21), the absence of a comprehensive and contemporaneous study based on robust data and methodologies currently hampers the elucidation of disease dynamics within the country and the identification of local risk factors.

We aim to provide a systematic analysis of the patterns of mortality, disability, and related risk factors in Peru over the past 30 years, prior to the COVID-19 pandemic, using estimates from the Global Burden of Disease (GBD) 2019 study. The production of this study was undertaken as a collaborative effort within the GBD Collaborator Network, adhering to the guidelines and protocols established by the GBD Protocol (22).

## 2. Methodology

### 2.1. Overview

The GBD study provides a comprehensive and systematic approach to quantify the magnitude of health loss worldwide using Bayesian methods (23). In the GBD 2019 study, the burden caused by 369 diseases and injuries, 87 risk factors, and 3,484 disease sequelae by sex and age was estimated for 204 countries and territories from 1990 to 2019. All measures are reported with 95% uncertainty intervals (UIs) corresponding to the 2.5^th^ and 97.5^th^ centile of the posterior distribution of 1,000 random draws. For comparative purposes, age-standardized rates are derived from the standard population structure developed for the GBD 2019 (23–25). All estimates are reported according to the Guidelines for Accurate and Transparent Health Estimates Reporting (Supplementary Table 1) (26). A comprehensive list of the input data sources and the code used to generate GBD 2019 estimates can be found on the Global Health Data Exchange, and results can be downloaded from the visualization tools (27, 28).

### 2.2. Data sources

Input data used in GBD 2019 to generate national estimates for Peru were retrieved from several sources, including the Institute of Statistics and Informatics of Peru (INEI, by its acronym in Spanish), the Ministry of Health, the National Prosecutor of the Public Ministry, the Ministry of Women and Vulnerable Populations, and the Institute of Health of Peru. Moreover, our analysis included data from five national censuses (1961 to 2017); vital registries (1950 to 2018); the Demographic and Health Survey (1986 to 2014); epidemiological surveillance of infectious diseases (1950 to 2017); administrative records (i.e., policy records, Peru National Program Against Domestic and Sexual Violence 1998–2015 [PNCVFS]), and a total of 146 surveys providing data on demographics, fertility, maternal and perinatal health, non-communicable diseases (NCDs), and risk factors (Supplementary Table 2).

### 2.3. Estimation of the population

The GDB demographic framework is an integrated and interconnected modeling process to estimate mortality, fertility, and population. A Bayesian hierarchical cohort component model (29) was used to estimate age-specific population and migration consistent with the estimates of age-specific fertility and mortality and the available census and registry data (24, 30). Then, the estimated age-specific fertility, mortality, migration, and baseline population were used to calculate consistent age-specific population estimates (24).

### 2.4. Estimation of fatal outcomes

All-cause and cause-specific mortality estimation methods used in GBD 2019 have been described previously (23, 24). Briefly, adult and under-5 mortality rates were analyzed with a multistage synthesis of all the available data. Data quality and completeness of the vital registration systems were used to estimate cause-specific mortality. Implausible causes of death were systematically redistributed to the most likely cause of death using GBD 2019 garbage code algorithms. Additional misclassifications due to HIV/AIDS and fatal discontinuities (natural disasters, major accidents, terrorism, etc.) were also corrected (23, 31).

Causes of death were modeled using the Cause of Death Ensemble model (CODEm) (32), which consists of an array of models informed by various mortality measures (number of deaths, cause fractions, and rates) and country-specific covariates. The final model was selected based on out-of-sample performance for root mean squared error (32). CoDCorrect was used to ensure consistency between the mortality envelope and the cause-specific mortality (23, 24).

Premature mortality was measured using years of life lost (YLLs), computed as the sum of each death subclassification group multiplied by the standard age-specific life expectancy, and these YLLs values were then summed to obtain the total YLLs for that specific group (23, 24).

### 2.5. Estimation of non-fatal outcomes and risk factors

To allow consistency between epidemiological parameters, we used DisMod-MR 2.1, an updated Bayesian-regression modeling tool, to analyze all available data on incidence, prevalence, remission, and others. Years lived with disability (YLDs) for sequelae were obtained by multiplying the prevalence of each sequela by the disability weight of the corresponding health state (33, 34). National–level disability-adjusted life years (DALYs) were computed by summing YLLs and YLDs for each cause, age, sex, and year from 1990 to 2019 (23). Risk-attributable fractions and their corresponding burden by cause were estimated following the GBD comparative risk assessment framework (25).

A recently developed decomposition method (25) was used to quantify the impact of population aging, demographic, and epidemiologic drivers on DALYs attributable to risk factors.

The socio-demographic index (SDI) was calculated as a composite indicator of development status that correlates strongly with health outcomes. The SDI includes three components: mean of lag-distributed income *per capita*, mean educational attainment for those aged 15 and older, and the total fertility rate in females under 25. To calculate the SDI, we first rescaled each component to obtain values between 0.005 to 1; then, we calculated the geometric mean of the three rescaled components. The resulting SDI ranges from 0 to 1, where higher values correspond to higher levels of development (24, 35). The outcomes in Peru were compared to those of countries with similar SDIs and/or geographic proximity in the Latin American (LA) region.

## 3. Results

### 3.1. Population

From 1990 to 2019, the population in Peru increased from 21.7 million to 33.9 million inhabitants (49.9% women). The population structure changed notably, from a predominantly young population in 1990 to a balanced group with both young and adults in 2019. During this period, the average annual population growth rate was 1.5%, and individuals aged 65 and older had the highest growth rate (3.6%) (Supplementary Figure 1).

### 3.2. Mortality

**Figure 1:**
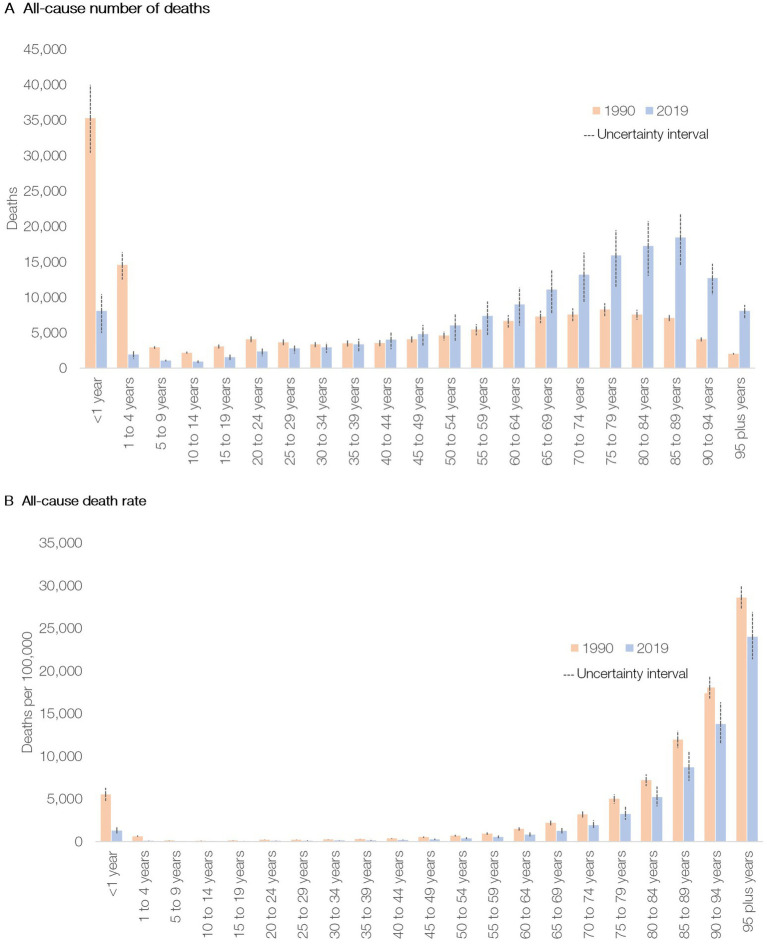
All-cause deaths **(A)** and mortality rate **(B)** in Peru by age group in 1990 and 2019.

The number of total deaths in Peru increased from 140,428 (95% uncertainty interval [UI], 129,556-152,158) in 1990 to 152,433 (118,652-194,458) in 2019. However, from 1990 to 2019, mortality rates (per 100,000 inhabitants) decreased among all age groups, dropping 16 and 80% in the population over 70 years and under-5 children, respectively (Figure 1; Supplementary Table 3). The cause-specific mortality distribution changed substantially during the 30-year analysis. NCDs accounted for 44.9% (42.7–47.2) of total deaths in 1990 and for 70.9% (69.3–73.2) in 2019. Communicable, maternal, neonatal, and nutritional (CMNN) diseases represented 40.7% (38.4–43.2) of total deaths in 1990 and 20.3% (18.2–21.8) in 2019. Deaths from injuries decreased from 14.4% (13.4–15.3) to 8.8% (8.2–9.4) from 1990 to 2019. While death rates due to CMNN diseases and injuries decreased by 65.5 and 57.4%, respectively, NCDs mortality rates increased by 9.6% over the same period (Supplementary Table 4).

From 1990 to 2019, life expectancy (LE) at birth increased from 71.6 (70.2–73.0) to 81.8 (79.0–84.4) years in women and from 67 (65.5–68.6) to 78.7 (75.3–82.0) years in men. The main drivers of LE gains were reductions in mortality due to respiratory infections and tuberculosis, ischemic heart disease (IHD) and stroke, maternal and neonatal conditions, and unintentional injuries (Supplementary Table 5).

Mortality time trends differed between age groups and sexes. Mortality rates of the top ten causes of death in under-5 children declined. The largest reductions were observed in lower respiratory infections (LRIs), diarrheal diseases, and protein-energy malnutrition. Mortality rates also decreased in the population aged 5–14 years. The leading causes of death in this age group were road injuries, LRIs, foreign body accidents, and leukemias (Supplementary Table 6).

In 2019, NCDs accounted for six of the ten leading causes of death among women aged 15–49, with cervical cancer, road injuries, and maternal disorders being the most lethal. Although death rates due to cervical (−23.1%) and breast cancer (−5.9%) declined between 1990 and 2019, cervical cancer escalated from the fifth cause of death in women to the first cause of mortality in women of reproductive age in this same period. Moreover, breast cancer climbed from the tenth to the seventh mortality cause in women aged 15–49. Among 50-69-year-old men, IHD, cirrhosis, LRIs, and Alzheimer represented the four highest causes of death among the top ten (Supplementary Table 6).

For the population over 70 years, the diseases that displayed increased death rates from 1990 to 2019 were interstitial lung diseases (female, 51%; male, 37%), chronic kidney disease (female, 43%; male, 14%), diabetes mellitus (DM) (female, 22%; male, 23%), and Alzheimer’s disease (female, 22%; male, 12%) (Supplementary Table 6).

### 3.3. Prevalence and incidence

The most prevalent diseases in 1990 were oral disorders (OD) (54.2% [49.1–59.0]), tuberculosis (29.7% [26.0–33.6]), and headache disorders (26.9% [24.0–29.7]). Similarly, in 2019, the three most prevalent diseases were OD (56.7% [52.2–61.1]), headache disorders (29.6% [26.6–32.7]), and sexually transmitted infectious diseases (excluding HIV) (27.2% [24.0–30.9]). From 1990 to 2019, the prevalence of tuberculosis and dietary iron deficiency decreased the most (at least 40%), while chronic kidney disease (27.4% [22–34.7]) and other musculoskeletal (MSK) (15.3% [8.2–22.8]) increased considerably (Supplementary Figure 2). In both 1990 and 2019, upper respiratory infections (48.0% [44.5–51.7] in 1990; 44.7% [41.6–48.0] in 2019), diarrheal diseases (13.0% [11.7–14.3] in 1990; 17.2% [15.6–18.9] in 2019), and OD (9.8% [8.4–11.2] in 1990; 8.9% [7.8–10.1] in 2019) had the highest incidence (Supplementary Figure 3).

### 3.4. Years lived with disability

**Figure 2:**
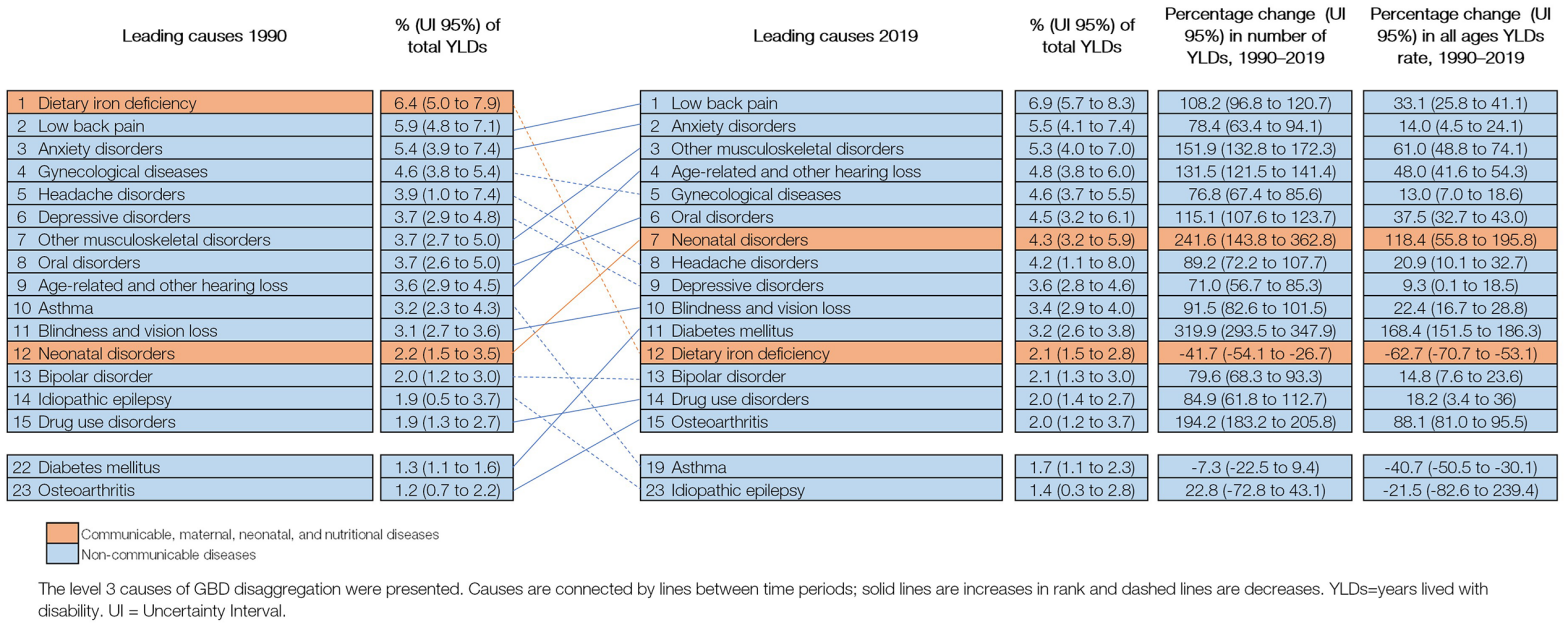
Leading 15 causes of YLDs in Peru (1990 and 2019), with change (%) in number of YLDs and all ages YLDs rate.

In 2019, the leading causes of YLDs in Peru were low back pain (6.9% of total YLDs [5.7–8.3]), anxiety disorders (5.5% [4.1–7.4]), other MSK (5.3% [4.0–7.0]), and age-related hearing loss (4.8% [3.8–6.0]). In 1990, the leading cause of YLDs was dietary iron deficiency (6.4% [5.0–7.9]), and by 2019 it dropped to the 12th place (2.1% [1.5–2.8]) (Figure 2).

### 3.5. Disability-adjusted life-years

In 2019, Peru reached 7.5 million (6.1–9.1) DALYs, of which 55.2% (51.2–60.1) were due to premature death and 44.8% (40.8–48.2) due to disability. In contrast, most DALYs (79.4% [79.1–80.1]) in 1990 were due to premature deaths (Supplementary Table 7).

**Figure 3:**
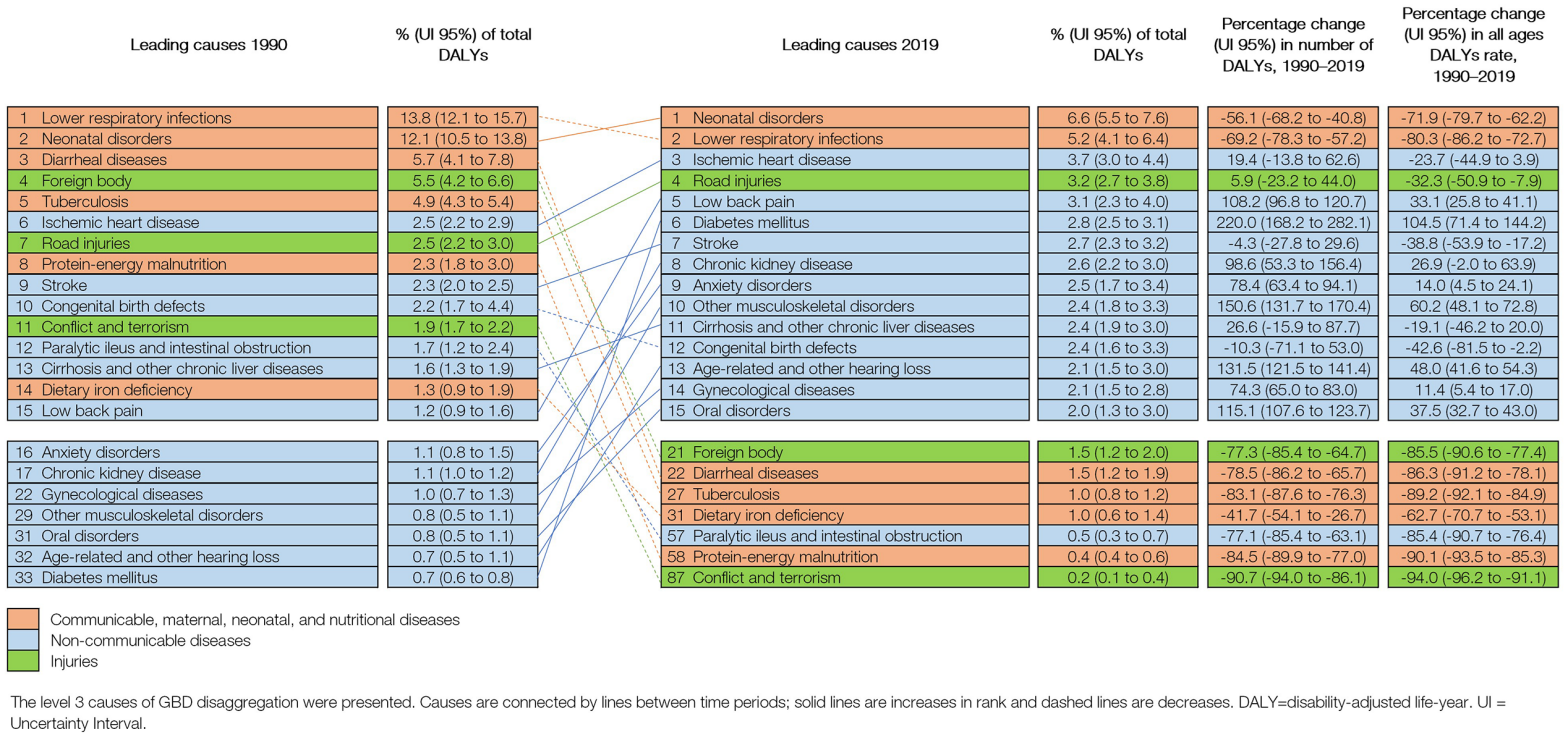
Leading 15 causes of DALYS in Peru (1990 and 2019), with change (%) in number of DALYS and all ages DALYs rate.

The distribution of 15 leading causes of DALYs changed significantly between 1990 to 2019. LRIs (13.8% of total DALYs [12.1–15.7] in 1990; 5.2% [4.1–6.4] in 2019) and neonatal disorders (12.1% [10.5–13.8] in 1990; 6.6% [5.5–7.6] in 2019) remained as the top leading causes of health loss although in a different order, and all-ages DALY rate due to both conditions decreased from 1990 to 2019. IHD (3.7% [3.0–4.4]), road injuries (3.2% [2.7–3.8]), and low back pain (3.1% [2.3–4.0]) became the third, fourth, and fifth causes of DALYs. DALYs due to NCDs increased between 1990 and 2019. During that period, the largest increases were observed in DM (104.5% [71.4–144.2]), other MSK (60.2% [48.1–7.8]), age-related and other hearing loss (48.3% [41.6–54.3]), and OD (37.5% [32.7–43.0]) (Figure 3).

### 3.6. Burden attributable to risks factors

From all DALYs in 1990 and 2019, 41.8% (39.5–44.3) and 33.8% (31.0–36.6), respectively, were attributable to exposure to all risk factors (Supplementary Table 8). The decrease in the absolute number of risk-attributable DALYs was driven by population aging (−16.8%), population growth (56.4%), risk exposure (−21.3%), and risk-deleted DALYs rate (−52.9%) (Supplementary Figure 4).

**Figure 4:**
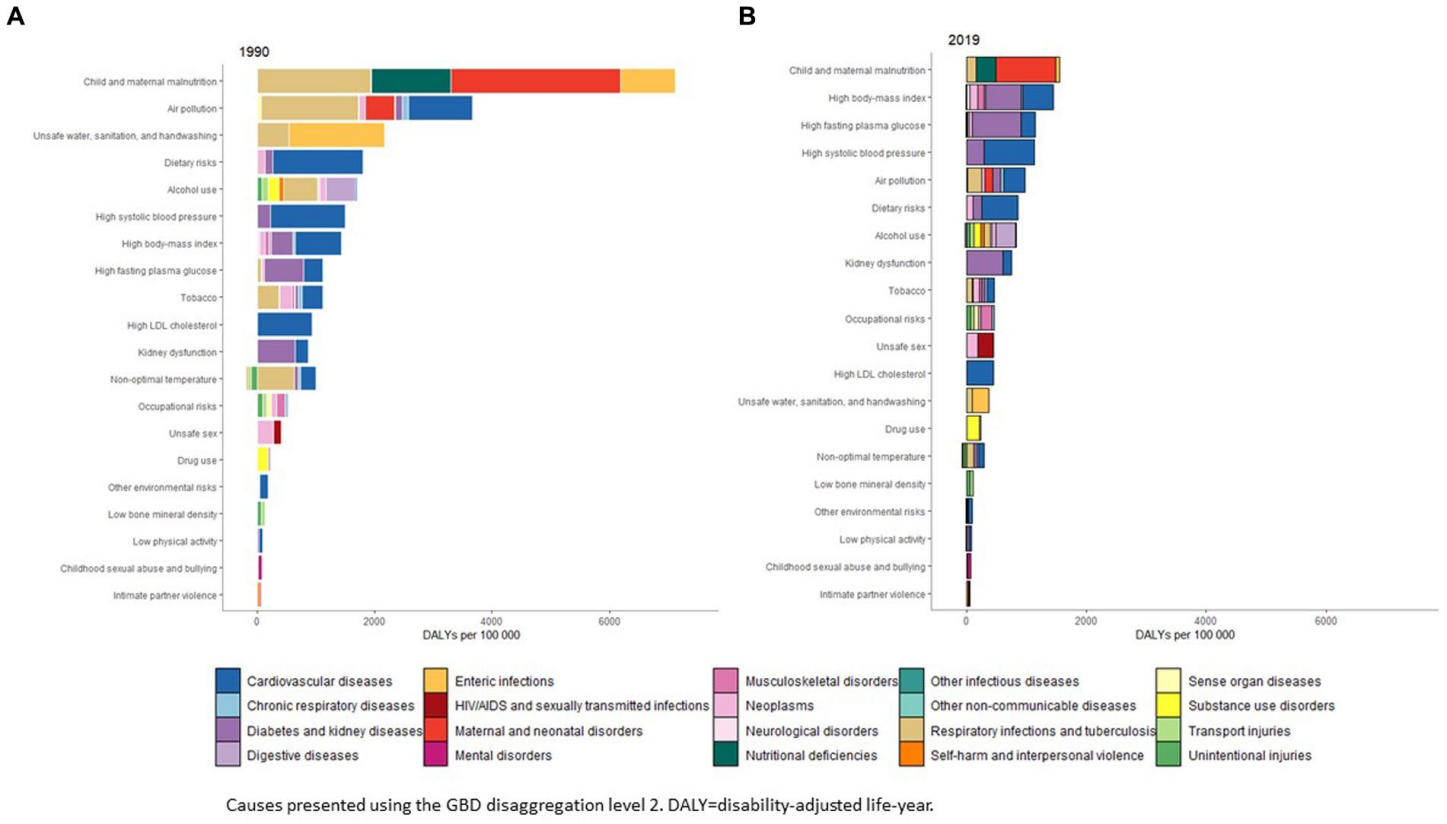
Age-standardized rates of DALYS attributable to risk factors in Peru in 1990 and 2019.

From 1990 to 2019, child and maternal malnutrition remained the primary risk factor for age-standardized DALYs rates, despite these rates being lower in 2019 (Figure 4).

The top 2, 3, and 4 risk factors attributable to DALYs in 2019 were high body-mass index (6.4% of total DALYs [4.2–8.7]), high fasting plasma glucose (5.1% [4.4–6.0]), and high systolic blood pressure (5.0% [4.1–5.9]) (Supplementary Table 8; Figure 4).

Supplementary Figure 5 shows the main risk factors for the four top causes of DALYs in 2019. The main risk factors for neonatal diseases were child and maternal malnutrition and air pollution. The top three risk factors for DALYs due to LRIs were air pollution, child and maternal malnutrition, and non-optimal temperatures. For IHD, the main risk factors were diet, high systolic blood pressure, and LDL cholesterol. Regarding road injuries, alcohol use was the most relevant risk factor.

### 3.7. 2019 DALYs rates in Peru in comparison with countries from the LA region

**Figure 5:**
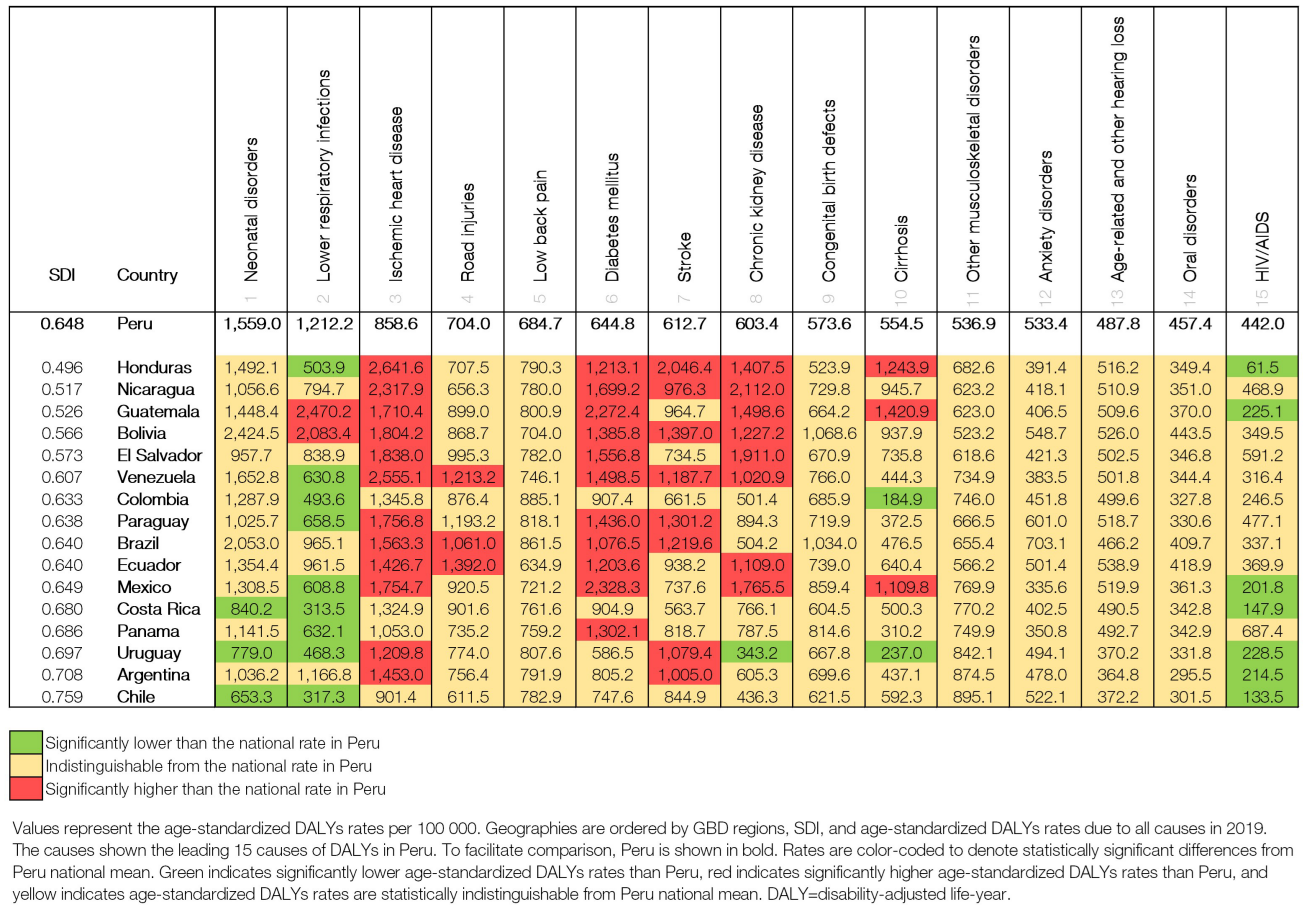
Age-standardized DALYS rates per 100,000 for Peru in comparison with Latin American countries in 2019.

We compared DALYs rates in Peru with 16 countries from LA with geographic proximity and similar SDIs. Figure 5 shows the top 15 causes of age-standardized 2019 DALYs rates for Peru and the countries sorted by SDI. DALYs rates due to LRIs were higher in Guatemala (2,470.1 [1,916.8-3,182.5]), Bolivia (2,083.4 [1,668.1-2,539.7]), and Peru (1,212.2 [893.1–1,616.5]). The DALYs rate due to HIV/AIDS in Peru (442 [258.6–751.2]) was significantly higher than those of Uruguay (228.5 [213.2–250.4]), Argentina (214.5 [189.9–253.7]), Chile (133.5 [120.6–158.3]), Costa Rica (147.9 [140.2–156.6]), and Mexico (201.8 [195.3–210.5]), Guatemala (225.1 [211.3–245.3]) and Honduras (61.5 [47.1–80.9]). Peru has significantly lower DALYs rates than other LA countries for IHD, DM, stroke, chronic kidney disease, and cirrhosis (Figure 5).

## 4. Discussion

Over the past three decades, Peru has undergone a significant demographic and epidemiological transition characterized by a multifaceted burden of disease encompassing select communicable diseases, NCDs, and injuries. Among these three categories, NCDs have emerged as the primary contributors to the overall disease burden in the country. While notable advancements have been observed in reducing mortality associated with LRIs, neonatal disorders, and diarrheal diseases in children under the age of five, the burden from conditions such as diabetes mellitus, MSK disorders chronic kidney disease, and mental disorders has increased. Conversely, the progress in terms of non-fatal outcomes, including oral disorders, headache disorders, sexually transmitted infectious diseases (excluding HIV), and upper respiratory infections have been limited or negligible.

Simultaneously, Peru has experienced a shift in the population structure transitioning from a predominantly young demographic in 1990 to a more balanced distribution that encompassed both young and adult age groups by 2019 with the age group of 65 years and older displaying the highest growth rate during the study period. At the global scale, Peru is one of the 11 countries with significant improvements in LE with a sustained 35-year increase since 1950 (36). This increase mainly reflects improvements in maternal and child health outcomes (37–39) and adult mortality due to infectious diseases. The mortality rate decreased consistently in the under-5-year group, which allowed one of the Millennium Development Goals (MDG4) to be achieved and set Peru on track to accomplish the Sustainable Development Goals (SDG 3.2) in 2030 (37). This progress may be associated to many factors, including the Peruvian economy, social improvement, civil society efforts, and government-led health and investment initiatives. Several government administrations introduced these investment initiatives as a response to a national commitment entitled the “National Agreement from the Roundtable for the Fight against Poverty” (Acuerdo Nacional de la Mesa de Concertación para la Lucha contra la Pobreza), which was signed by key actors of the society, including political parties and the civil society (40). Other efforts that most likely contributed to maternal, neonatal, and child health were vaccination programs, nutrition assistance for the poorest population, the Integrated Management of Childhood Illness Program (41), improved sanitation facilities, and the promotion of handwashing and drinking water sources (7, 37, 42).

Despite a reduction in neonatal mortality observed during the study period, our results indicate that neonatal disorders emerged as the main cause of health loss in Peru in 2019, consistent with data estimates from the Pan American Health Organization (PAHO) (43). The major contributors to neonatal deaths in Peru include prematurity-immaturity, infections, and asphyxia (37, 44).

Previous studies have shown that approximately half of the neonatal deaths occurred in hospitals on the coast of Peru, particularly in Lima, where most referral hospitals are located. In contrast, most of the neonatal mortality observed in the Peruvian highlands and Amazon jungle occurred in home settings (44). Also, higher neonatal mortality was found in departments with greater poverty and less education (45). In order to effectively confront this scenario, policies aimed at addressing and preventing neonatal mortality must encompass considerations of geographical gaps, as well as cultural and educational disparities. This comprehensive approach would facilitate improved and timely access to healthcare services.

LRIs imposed one of the major burdens on the healthcare system (46). In 1990, LRIs were the main cause of DALYs in Peru. However, by 2019, while remaining the second cause of health loss, there was a substantial 80% reduction in the absolute number of DALYs attributed to LRIs. This decrease was achieved by improved access to medical care, higher availability of antibiotics, and the implementation of Hib pneumococcal conjugate vaccines (47). Compared to other LA countries, Peru, along with Guatemala and Bolivia, exhibited the highest DALYs due to LRIs.

The distribution of LRIs and other respiratory infections is not the same throughout Peru. A study that compared the incidence of acute respiratory infections between geographic regions in under-5 children showed higher rates in the Peruvian Amazon (15.4%) and the Cost region (15.2%) than in the Andean region (13.9%) (48). The factors contributing to higher pneumonia mortality in certain regions include delayed diagnosis and treatment (due to unrecognized signs and symptoms), self-medication, and lack of access to health services, equipment, and supplies like antibiotics, oxygen, and nebulization (49). Together with other social determinants like poverty and overcrowding, the same factors can probably explain Peru’s high mortality during the COVID-19 pandemic (50–52). In this complicated context, the lack of oxygen was the main factor documented at global, regional, and local levels (53–55). Two key aspects underlie this crisis in Peru: the oxygen market was controlled by only two global companies, and the prices were previously agreed (55–57). This situation was created because of a law in 2010 (58) and was only changed recently (59). Importantly, the MoH must design and implement actions to address the structural and cultural factors that delay diagnosis and treatment, especially in the regions with a higher burden of respiratory diseases. Furthermore, medical oxygen systems should be well-designed and equally available for all. New policies should reduce access gaps and design strategies to reduce exposure to the risk factors identified in this study, such as air pollution and child and maternal undernutrition. Better ventilatory standards for buildings, schools, and transportation are also very much needed.

Our study evidenced significant progress in the control of tuberculosis, achieved by the Peruvian National Health Strategy for the Prevention and Control of Tuberculosis (NHSPCT) and other national strategies (7, 60, 61). Nevertheless, tuberculosis was still within the top ten prevalent diseases in 2019; Peru has the second highest tuberculosis burden in the Americas and increasing cases of multi-drug-resistant tuberculosis (MDR-TB) (62, 63). Of all the tuberculosis patients in the world, 4.6% have MDR-TB, and this proportion has increased since 1990 (64). Few studies have explored possible causes for the high rates of MDR-TB. Managing MDR-TB is challenging, and medication adherence is a huge problem. Social determinants like poverty and poor healthcare access are critical contributors to the high MDR-TB rates (61–63). A qualitative study in the Peruvian jungle found three types of factors influencing the outcome of MDR-TB patients: individual factors, including myths, misbeliefs about medications, and stigma; external factors, such as the financial impact of MDR-TB on patients and their families; and mistrust in healthcare systems (65). Peru is still unable to fully control tuberculosis despite several national efforts like the Prevention and Control of Tuberculosis law (60), which supports the activities of the National Program.

Peru’s third most prevalent condition in 2019 was sexually transmitted infectious diseases (excluding HIV), which represent a global and regional public health challenge (66). The country must warrant more attention and health prevention services to high-risk populations and the general public (67, 68).

In Peru, NCDs represent a significant burden of disease, with seven out of the top ten causes of death in adults in 2019 associated with NCDs. Notably, cervical cancer was the leading cause of death among women of reproductive age. The Peruvian Ministry of Health (MoH) launched initiatives to prevent gynecological cancer more than two decades ago. In 2011, the MoH introduced the human papillomavirus vaccination (HPV) for girls (69), reaching a coverage close to 78% in 2019 for two doses. However, the pandemic significantly affected all the country’s vaccination programs, and HPV vaccine rates will probably drop in the following years (70). In 2012, the Peruvian Government launched the “Plan Esperanza,” which aimed to provide screening for breast, cervical, stomach, and prostate cancer in healthcare establishments nationwide (71). However, multiple factors hinder opportunities for screening, and in the case of cervical cancer, almost half of the women are diagnosed in the late stages, resulting in high mortality (72, 73). National strategies to introduce HPV testing and promote self-sampling (to increase coverage) are imperative to ensure HPV (+) women are promptly treated and continuously cared for.

IHD was found to be one of the top ten causes of mortality in men over 50 years old in 2019, representing an important shift from 1990. Furthermore, compared with other countries in LA with similar SDI, Peru has a lower IHD DALYs rate, probably associated with the late introduction of ultra-processed food (74, 75). DM burden in Peru has doubled in the past 30 years, and, similar to IHD, DM burden is around the lowest compared to 16 other countries in the LA region. However, most risk factors associated with IHD and DM have increased in Peru from 1990 to 2019, probably due to the country’s rapid urbanization and the development of an “obesogenic” environment (76).

In 2013, Peru enacted the Law for the Promotion of Healthy Eating in Boys, Girls, and Adolescents (La Ley N° 30,021, Ley de Promoción de la Alimentación Saludable para Niños, Niñas y Adolescentes) (77). However, it was not until 2017 that the law was fully implemented. This legislation mandates the introduction of food labels indicating high levels of added sugars, added saturated fats, added salt, and trans-fat, empowering the citizens to make better-informed food choices and reduce the consumption of processed foods which have been associated with the increased burden of NCDs. Recent reports indicate that labeled products have been consumed approximately 4% less (78). Furthermore, in order for these initiatives to be effectively implemented, it is crucial that they consider the contextual factors at play. The ongoing implementation of these laws necessitates further research to evaluate their impact on diverse populations. Additionally, the National Agenda should address remaining priorities, such as enhancing primary care services for NCDs (including IHD and DM), ensuring timely diagnosis and appropriate health management, and conducting comprehensive behavioral interventions aimed at reducing risk factors at the population level. These actions will contribute to a more comprehensive and impactful approach to public health.

Although oral disorders (OD) are highly prevalent worldwide, oral health is a neglected area of global health (23). Most countries do not measure or include OD in their national health surveys and agendas, so policymakers do not consider them (79, 80). Peru began a program to add fluoride to drinking water in 1950, followed by the addition of fluoride in salt (1984) and milk (1999) (81), but according to our data, more than half of Peruvians have been affected by OD in the last 30 years. Compared to other regions in the world, the LA region has a higher prevalence and incidence of untreated cavities in permanent teeth, severe periodontal diseases, and tooth loss; this is also the case in Peru (82). To address OD, the World Health Organization (WHO) encourages nations to shift from a conventional to a preventive model and to include oral health as NCD and as part of the Universal Health Coverage (UHC) agenda (83, 84). Consequently, the importance of OD should be highlighted nationally.

Headache disorders are among the most prevalent and disabling conditions globally (85, 86), and they impose a high cost on society (87). In Peru, headache disorders were the second most prevalent cause in 2019. Currently, there is still little information available on this subject. In general, headache disorders are underestimated and underdiagnosed, so more population-based and disease-specific surveys are needed to address these matters and obtain accurate estimates for designing better interventions.

Our data show that MSK, including low back pain (LBP), anxiety disorders, and age-related hearing loss are the top causes of YLDs. MSK diseases, although generally non-lethal, impose a considerable economic burden on patients and the healthcare system (23, 88). In 2019, LBP was one of the top causes of DALYs in Peru, and globally, it remains the leading cause of disability and absenteeism from work (89, 90). Other studies have shown that the medical and economic burden due to LBP will increase significantly in the following decades due to the demographic transition, obesity, and physical inactivity; moreover, this increase will be higher among low-income and middle-income countries (91, 92). Peru urgently needs effective programs and policies to prevent disability and provide adequate care and rehabilitation for LBP, mental conditions, and hearing loss, especially among vulnerable people. This will require policymakers to recognize that unless we address these conditions leading to disability, the investments resulting in longer lifespans will be wasted due to the loss of quality of life.

In 2019, road injuries were the fourth cause of DALYs and one of the main causes of death in people between 5 and 49 years old, even though the countries with the highest-burden due to road injuries in LA are Venezuela, Brazil, and Ecuador. Pedestrians (23%), motorcyclists (23%), and cyclists (3%) are the most frequent victims of road traffic deaths in the Americas; in some parts of Peru, the number of pedestrian victims reaches 80% (93, 94). These deaths are related to different risk factors (speed, drunk driving, knowledge of traffic signs or illiteracy, fatigue, failure to use motorcycle helmets, seatbelts, and child restraints) and specific city-level attributes (94, 95). Globally, road injury mortality has improved in the last decades; in Latin America, cities with higher population density, higher intersection density, and a mass transit system such as a subway or bus rapid transit system had significantly lower road-traffic death rates than cities without these characteristics (95). Urban landscape, street network design, and public transportation policies can affect a whole city in the long term (95). We recommend that policies align with the global targets of road safety intervention to prevent injuries and deaths, including stakeholders from all sectors involved. Furthermore, policies should strengthen some key points regarding the enforcement of safety laws, the safety of vulnerable road users, and traffic management. These initiatives must be acknowledged as components within a comprehensive framework, with a clear understanding of their role as a specific tool in addressing this public health issue at hand.

In Peru, dietary iron deficiency was the leading cause of DALYs in 1990; by 2019, it had decreased by 62.7%. Iron deficiency is the most common cause of anemia, which is still highly prevalent in Peru; in 2009 and 2017, anemia prevalence was 50.4 and 43.6%, respectively, in under-3 children (96). Reducing anemia has become a high priority of the Peruvian National Government, which has performed interventions such as the distribution of free multi-micronutrient supplements and educational campaigns to promote the consumption of iron-rich foods (7, 96, 97). To further address the anemia in the country, interventions must go beyond dietary practices and consider the social determinants affecting population health in diverse regions.

This study has several limitations related to the GBD 2019 methodology. First, the precision of the estimations can be affected by time lags in the available data, country-level aggregate data, the absence of data from age groups and time periods, and the unreliability of the available data—for example, the estimation of respiratory infection, which was estimated from data from a younger population. Second, despite the reduction of deaths assigned to the garbage code, the estimated time trends might still be affected. Third, most national surveys are focused on CMNN and some NCDs, leaving out diseases that saturate the healthcare system, such as MSK and mental diseases. Furthermore, the nature of nationally representative surveys or administrative data is restricted among age groups, and for the geographical areas with the highest prevalence or mortality levels, this can also affect the precision of estimations. Finally, efforts should be directed to integrate the administrative records of an information system to fill data gaps, avoid duplicate registries, and have information available at the individual level. In order to validate GBD estimates, they should be critically reviewed and compared with local reports.

## 5. Conclusion

Despite facing challenges in health investment and experiencing political, economic, and social instability, Peru has achieved significant progress in reducing mortality rates between 1990 and 2019, particularly in premature mortality caused by communicable diseases. However, these reductions in mortality are not uniform across diseases and age groups. A notable concern is the increasing burden of disability resulting from non-communicable diseases (NCDs). To address this complex epidemiological profile, Peru needs to invest in the redesign and strengthening of its healthcare system, as well as improving health information systems, which are crucial for guiding effective policies.

It is important to recognize that the overall trends and disease burden at the national level may conceal existing disparities at the subnational level. Therefore, future research should focus on evaluating subnational estimates to gain a more nuanced understanding of the country’s health challenges.

Furthermore, the pre-existing advancements in health metrics achieved in Peru prior to the COVID-19 pandemic are now under threat due to the ongoing global health crisis. As a result, it is crucial for the government to proactively address the emerging healthcare demands posed by the pandemic and adapt strategies accordingly.

In summary, Peru has made significant strides in improving health outcomes, but there are still substantial challenges to overcome, particularly in addressing the burden of NCDs and ensuring equitable healthcare access. By investing in healthcare system strengthening, enhancing health information systems, and taking proactive measures in response to the COVID-19 pandemic, Peru can continue its progress toward achieving better health for its population.

## Data availability statement

The original contributions presented in the study are included in the article/Supplementary material, further inquiries can be directed to the corresponding author.

## Author contributions

NA, LA, MA-B, BAQ, CPB, EB, AKD, TF, VKG, KK, VCL, SL, RL, RM, WM, AHM, EM, CJLM, BTN, OCO, VP-R, CR, MJR-B, VDR, IZ, CC, and PJG: providing data or critical feedback on data sources. LA and AHM: developing methods or computational machinery. NA, DSA, JEA-R, LA, BAQ, FJB, CPB, EB, LC-H, RC, PRC-U, SC, CCH, AKD, TGGD, TF, MG, VKG, SIH, BYH-S, HK, KK, AK, VCL, SL, RM, WM, AHM, EM, EM-Z, CJLM, BTN, MN, OCO, DVO-A, AO, KP-B, VP-R, IQ, CR, MJR-B, VDR, MS, JWS, SSA, DASS, DV, BW, NDW, AY, CC, and PJG: providing critical feedback on methods or results. NA, DSA, LA, MA-B, BAQ, FJB, CPB, LC-H, PRC-U,JC-C, CC, AKD, TGGD, NF, TF, MG, VVG, SIH, HK, KK, AK, FK, RL, WM, AHM, EM, EM-Z, CJLM, BTN, OCO, DO-A, KP-B, VP-R, IQ, CR, EMMR, MJR-B, VDR, SAS, DASS, DV, BW, NDW, AY, IZ, CC, and PJG: drafting the work or revising is critically for important intellectual content. SIH, AHM, VP-R, MJR-B, PJG, and CJLM: managing the estimation or publications process. All authors contributed to the article and approved the submitted version.

## Funding

BAQ acknowledges support from Belgian Directorate of Development Cooperation (DGD) through the Framework Agreement between the Belgian DGD and the Institute of Tropical Medicine, Belgium; Fogarty International Center of the National Institutes of Health and the University of California Global Health Institute under Award Number D43TW009343; and Fogarty International Center, and National Institute of Child Health & Human Development of the National Institutes of Health under award number D43 TW009763. PRC-U, acknowledges support from training grant D43 TW007393 awarded by the Fogarty International Center of the US National Institutes of Health. SC acknowledges support from Fondo de Financiamiento de Centros de Investigacion en Areas Prioritarias (FONDAP) (grant number 15130011). This study was also supported by the Bill & Melinda Gates Foundation.

## Conflict of interest

CB participation on a data safety monitoring board for COVID-out: Metformin and other COVID treatment study and is a member of the American Heart Association Epidemiology Leadership committee, outside the submitted work. LC-H is an employee of the Instituto Nacional de Salud Pública, Mexico and Universidad Peruana Cayetano Heredia. S Cortes reports support for the present manuscript from Fondo de Financiamiento de Centros de Investigacion en Areas Prioritarias (FONDAP) (grant number 15130011). VG reports grants or contracts from the National Health and Medical Research Council (NHMRC), Australia, paid directly to their institution, outside the submitted work. FK reports grants or contracts from Belgian Directorate of Development Cooperation (DGD) through the Framework Agreement between the Belgian DGD and the Institute of Tropical Medicine, Belgium; Fogarty International Center of the National Institutes of Health and the University of California Global Health Institute under Award Number D43TW009343; Fogarty International Center, and National Institute of Child Health & Human Development of the National Institutes of Health under Award Number D43 TW009763, outside the submitted work. WM is a United Nations Population Fund staff at the Peru Country Office, which does not necessarily endorse these results. JM reports grants from Alliance for Health Policy and Systems Research (HQHSR1206660), Bloomberg Philanthropies (grant 46129, via University of North Carolina at Chapel Hill School of Public Health), FONDECYT via CIENCIACTIVA/CONCYTEC, British Council, British Embassy and the Newton-Paulet Fund (223-2018, 224-2018), DFID/MRC/Wellcome Global Health Trials (MR/M007405/1), Fogarty International Center (R21TW009982, D71TW010877, R21TW011740), Grand Challenges Canada (0335–04), International Development Research Center Canada (IDRC 106887, 108167), Inter-American Institute for Global Change Research (IAI CRN3036), National Cancer Institute (1P20CA217231), National Heart, Lung and Blood Institute (HHSN268200900033C, 5U01HL114180, 1UM1HL134590), National Institute of Mental Health (1U19MH098780), Swiss National Science Foundation (40P740-160366), UKRI BBSRC (BB/T009004/1), UKRI EPSRC (EP/V043102/1), UKRI MRC (MR/P008984/1, MR/P024408/1, MR/P02386X/1), Wellcome (074833/Z/04/Z, 093541/Z/10/Z, 103994/Z/14/Z, 107435/Z/15/Z, 205177/Z/16/Z, 214185/Z/18/Z, 218743/Z/19/Z) and the World Diabetes Foundation (WDF15-1224), paid to their institution, and contracts from Health Action International; unpaid participation on data safety monitoring board, Nigeria Sodium Study (NaSS); Trial Steering Committee, INTEnsive care bundle with blood pressure Reduction in Acute Cerebral haemorrhage Trial (INTERACT 3); International Advisory Board, Latin American Brain Health institute (BrainLat), Universidad Adolfo Ibáñez (Chile); Consultative Board, Programa de Gastronomía, Facultad de Estudios Interdisciplinarios, Pontificia Universidad Católica del Perú; and Advisory Board, InterAmerican Heart Foundation (IAHF); and is the co-chair of the Independent Group of Scientists (IGS), 2023 Global Sustainable Development Report, United Nations; a is member of the Scientific Expert Committee, Global Data Collaborative for CV Population Health, World Health Federation, Microsoft, and Novartis Foundation; the Scientific and Technical Advisory Committee (STAC), Alliance for Health Policy and Systems Research, World Health Organization; the WHO Technical Advisory Group on NCD-related Research and Innovation (TAG/RI), Noncommunicable Diseases Department, World Health Organization; and the Advisory Scientific Committee, Instituto de Investigación Nutricional (Peru), all unpaid, outside the submitted work. DV reports scholarship support from the Canadian Institutes of Health Research and is an unpaid board member for the Global Alliance for Rheumatic and Congenital Hearts, all outside the submitted work. IZ was employed by Sina Medical Biochemistry Technologies Co. Ltd.

The remaining author declares that the research was conducted in the absence of any commercial or financial relationships that could be construed as a potential conflict of interest.

## Publisher’s note

All claims expressed in this article are solely those of the authors and do not necessarily represent those of their affiliated organizations, or those of the publisher, the editors and the reviewers. Any product that may be evaluated in this article, or claim that may be made by its manufacturer, is not guaranteed or endorsed by the publisher.

## Supplementary material

The Supplementary material for this article can be found online at: https://www.frontiersin.org/articles/10.3389/fpubh.2023.1189861/full#supplementary-material
