# Supplementary material for: Estimating mortality and disability in Peru before the COVID-19 pandemic: a systematic analysis from the Global Burden of the Disease Study 2019
Source: Front Public Health. 2023 Jun 22;11:1189861. doi: 10.3389/fpubh.2023.1189861 (PMC10325574; doi:10.3389/fpubh.2023.1189861)

Supplementary figures

Supplementary Figure 1. Population pyramid from Peru in 1990 and 2019

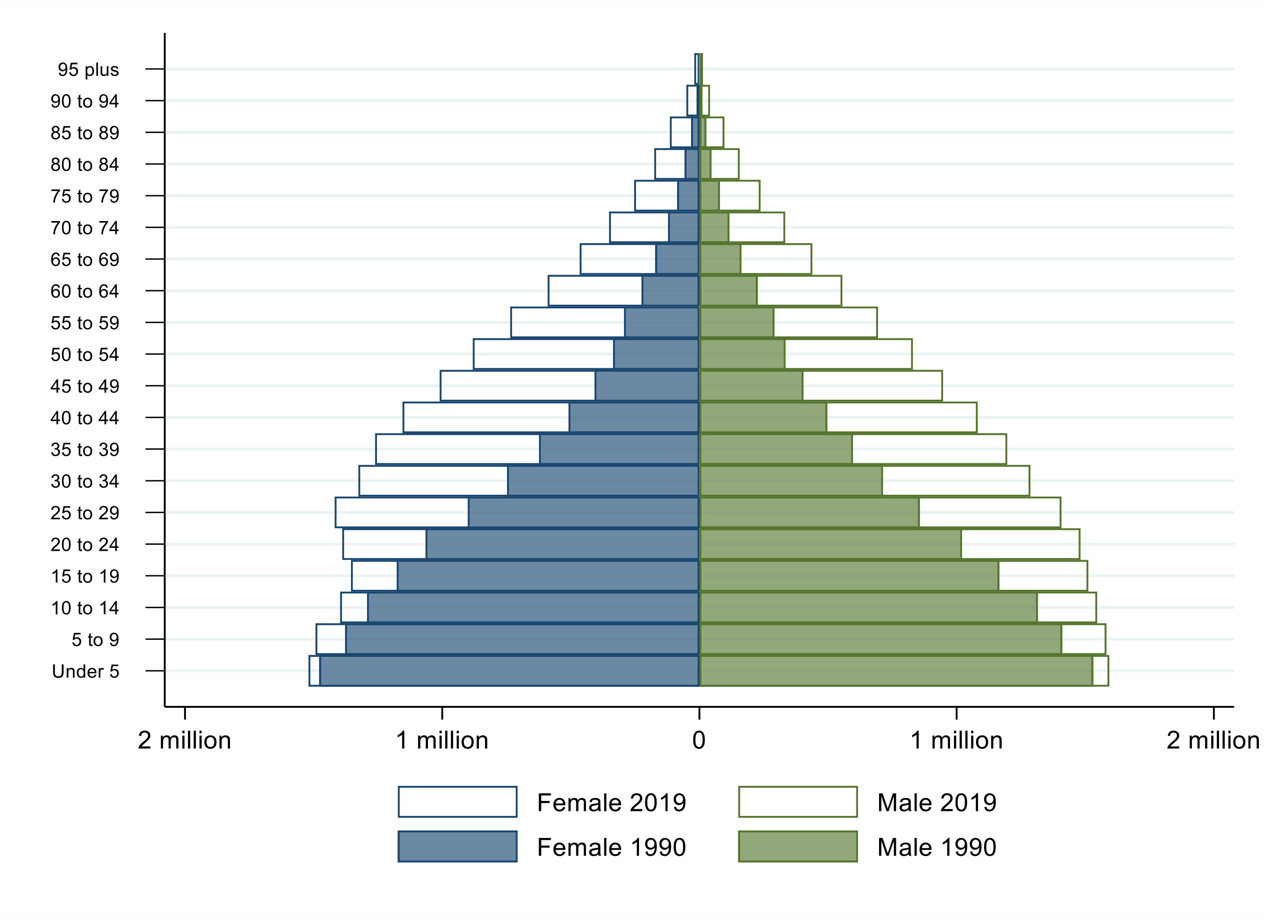

Supplementary Figure 2. Top 15th leading causes of prevalent cases in Peru (1990 and 2019), with change (%) in the number of prevalent cases and all-ages prevalence.

Leading causes 1990	% of total prevalent cases
1 Oral disorders	54·2 (49·1 to 59·0)
2 Tuberculosis	29·7 (26·0 to 33·6)
3 Headache disorders	26·9 (24·0 to 29·7)
4 Sexually transmitted infections excluding HIV	21·1 (19·3 to 22·9)
5 Gynecological diseases	19·7 (18·1 to 21·4)
6 Dietary iron deficiency	19·7 (17·8 to 21·7)
7 Hemoglobinopathies and hemolytic anemias	16·6 (15·1 to 18·1)
8 Age-related and other hearing loss	13·9 (13·1 to 14·8)
9 Intestinal nematode infections	13·0 (9·9 to 17·1)
10 Upper digestive system diseases	12·4 (11·0 to 13·7)
11 Fungal skin diseases	11·7 (10·2 to 13·2)
12 Cirrhosis and other chronic liver diseases	8·6 (7·8 to 9·5)
13 Asthma	7·4 (5·8 to 9·4)
14 Other skin and subcutaneous diseases	6·8 (6·6 to 7·0)
15 Blindness and vision loss	6·5 (5·7 to 7·3)
16 Chronic kidney disease	5·3 (4·3 to 6·8)
23 Other musculoskeletal disorders	4·0 (3·3 to 4·7)

Leading causes 2019	% of total prevalent cases	Percentage change in number of prevalent cases, 1990–2019	Percentage change in all ages prevalence, 1990–2019
1 Oral disorders	56·7 (52·2 to 61·1)	63·1 (57·6 to 69·1)	4·2 (0·8 to 8·1)
2 Headache disorders	29·6 (26·6 to 32·7)	72·1 (65·0 to 79·9)	10·0 (5·5 to 15·0)
3 Sexually transmitted infections excluding HIV	27·2 (24·0 to 30·9)	101·3 (87·7 to 115·8)	28·7 (20·0 to 37·9)
4 Gynecological diseases	23·1 (21·4 to 24·8)	82·6 (77·0 to 88·3)	16·7 (13·1 to 20·4)
5 Age-related and other hearing loss	21·0 (19·8 to 22·3)	134·8 (128·8 to 141·7)	50·1 (46·3 to 54·5)
6 Hemoglobinopathies and hemolytic anemias	18·2 (16·2 to 20·4)	71·9 (65·8 to 78·1)	9·9 (6·0 to 13·9)
7 Upper digestive system diseases	16·3 (14·6 to 17·9)	105·5 (100·0 to 110·6)	31·4 (27·9 to 34·6)
8 Tuberculosis	14·2 (12·5 to 16·2)	-25·6 (-33·2 to -16·5)	-52·4 (-57·3 to -46·6)
9 Fungal skin diseases	13·3 (11·9 to 15·1)	78·4 (71·6 to 85·5)	14·1 (9·7 to 18·6)
10 Cirrhosis and other chronic liver diseases	13·1 (11·9 to 14·3)	137·9 (129·3 to 146·2)	52·1 (46·5 to 57·4)
11 Dietary iron deficiency	10·8 (9·4 to 12·2)	-14·3 (-27·3 to 1·2)	-45·2 (-53·5 to -35·3)
12 Chronic kidney disease	10·0 (8·3 to 12·3)	192·9 (174·7 to 212·8)	87·2 (75·6 to 100·0)
13 Blindness and vision loss	9·1 (7·9 to 10·5)	120·2 (110·4 to 130·4)	40·8 (34·5 to 47·3)
14 Other skin and subcutaneous diseases	8·7 (8·4 to 9·0)	100·7 (97·1 to 104·4)	28·3 (26·0 to 30·6)
15 Other musculoskeletal disorders	6·5 (5·5 to 7·5)	152·6 (135·4 to 171·8)	61·5 (50·5 to 73·7)
17 Intestinal nematode infections	6·2 (4·6 to 8·1)	-26·2 (-51·1 to 8·7)	-52·8 (-68·7 to -30·5)
23 Asthma	4·4 (3·3 to 5·9)	-7·1 (-22·8 to 9·7)	-40·6 (-50·6 to -29·9)

Communicable, maternal, neonatal, and nutritional diseases
Non-communicable diseases

The level-3 causes of GBD disaggregation are presented. Lines between time periods connect causes; solid lines represent increases in rank, and dashed lines represent decreases.

Supplementary Figure 3. Top 15th leading causes of incident cases in Peru (1990 and 2019), with change (%) in the number of incident cases and all ages-incidences

Leading causes 1990	% of total incident cases
1 Upper respiratory infections	48·0 (44·5 to 51·7)
2 Diarrheal diseases	13·0 (11·7 to 14·3)
3 Oral disorders	9·8 (8·4 to 11·2)
4 Fungal skin diseases	4·5 (3·9 to 5·1)
5 Bacterial skin diseases	3·0 (2·7 to 3·2)
6 Urinary diseases and male infertility	2·0 (1·7 to 2·3)
7 Lower respiratory infections	1·9 (1·7 to 2·1)
8 Scabies	1·7 (1·5 to 2·1)
9 Gynecological diseases	1·7 (1·4 to 1·9)
10 Headache disorders	1·4 (1·2 to 1·6)
11 Sexually transmitted infections excluding HIV	1·4 (1·2 to 1·7)
12 Other skin and subcutaneous diseases	1·1 (1·0 to 1·2)
13 Otitis media	1·0 (0·6 to 1·4)
14 Upper digestive system diseases	0·9 (0·7 to 1·0)
15 Dermatitis	0·8 (0·7 to 0·9)

Leading causes 2019	% of total incident cases	Percentage change in number of incident cases, 1990–2019	Percentage change in all ages incidence, 1990–2019
1 Upper respiratory infections	44·7 (41·6 to 48·0)	46·6 (38·7 to 55·3)	-6·3 (-11·3 to -0·7)
2 Diarrheal diseases	17·2 (15·6 to 18·9)	109·1 (92·7 to 126·2)	33·6 (23·2 to 44·6)
3 Oral disorders	8·9 (7·8 to 10·1)	43·6 (36·5 to 52·2)	-8·2 (-12·8 to -2·7)
4 Fungal skin diseases	5·1 (4·5 to 5·8)	79·1 (71·4 to 86·9)	14·5 (9·6 to 19·4)
5 Bacterial skin diseases	3·1 (2·9 to 3·3)	64·7 (60·4 to 68·9)	5·3 (2·5 to 7·9)
6 Urinary diseases and male infertility	2·4 (2·1 to 2·8)	88·2 (70·2 to 110·2)	20·3 (8·8 to 34·3)
7 Gynecological diseases	1·9 (1·6 to 2·2)	80·9 (74·5 to 86·7)	15·6 (11·5 to 19·3)
8 Sexually transmitted infections excluding HIV	1·6 (1·4 to 1·9)	80·5 (73·0 to 87·6)	15·3 (10·6 to 19·9)
9 Lower respiratory infections	1·6 (1·4 to 1·7)	34·6 (24·9 to 45·1)	-14·0 (-20·2 to -7·3)
10 Scabies	1·6 (1·3 to 1·8)	42·2 (36·1 to 48·5)	-9·1 (-13·0 to -5·1)
11 Headache disorders	1·4 (1·3 to 1·6)	58·7 (49·9 to 67·0)	1·4 (-4·2 to 6·7)
12 Other skin and subcutaneous diseases	1·4 (1·3 to 1·5)	99·5 (95·9 to 103·1)	27·5 (25·2 to 29·8)
13 Upper digestive system diseases	1·1 (1·0 to 1·2)	101·1 (95·3 to 106·4)	28·6 (24·8 to 31·9)
14 Dermatitis	0·9 (0·7 to 1·0)	79·2 (72·9 to 85·3)	14·5 (10·5 to 18·4)
15 Otitis media	0·7 (0·5 to 1·0)	14·0 (10·8 to 18·9)	-27·2 (-29·1 to -24·0)

Communicable, maternal, neonatal, and nutritional diseases
Non-communicable diseases

The level-3 causes of GBD disaggregation are presented. Lines between time periods connect causes; solid lines represent increases in rank, and dashed lines represent decreases.

Supplementary Figure 4. Percentage change in the absolute number of risk-attributable DALYs in Peru from 1990-2019 due to population growth, population aging, changes in exposure to each GBD risk factor, and changes in risk-deleted DALY rates for both sexes and all causes combined.

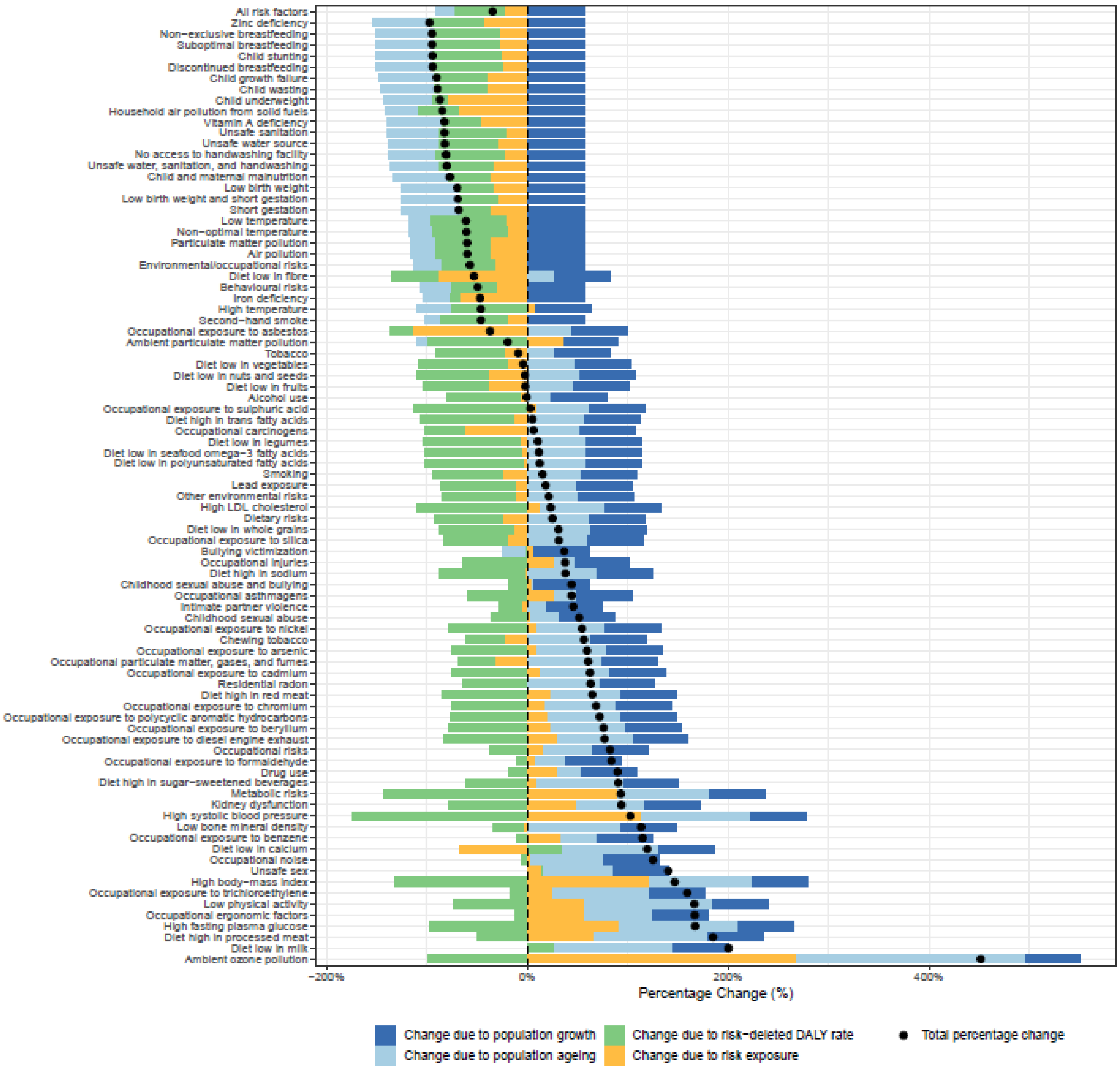

Supplementary Figure 5. Age-standardized rate of top four causes of DALYs attributable to risk factors in Peru in 1990 and 2019.

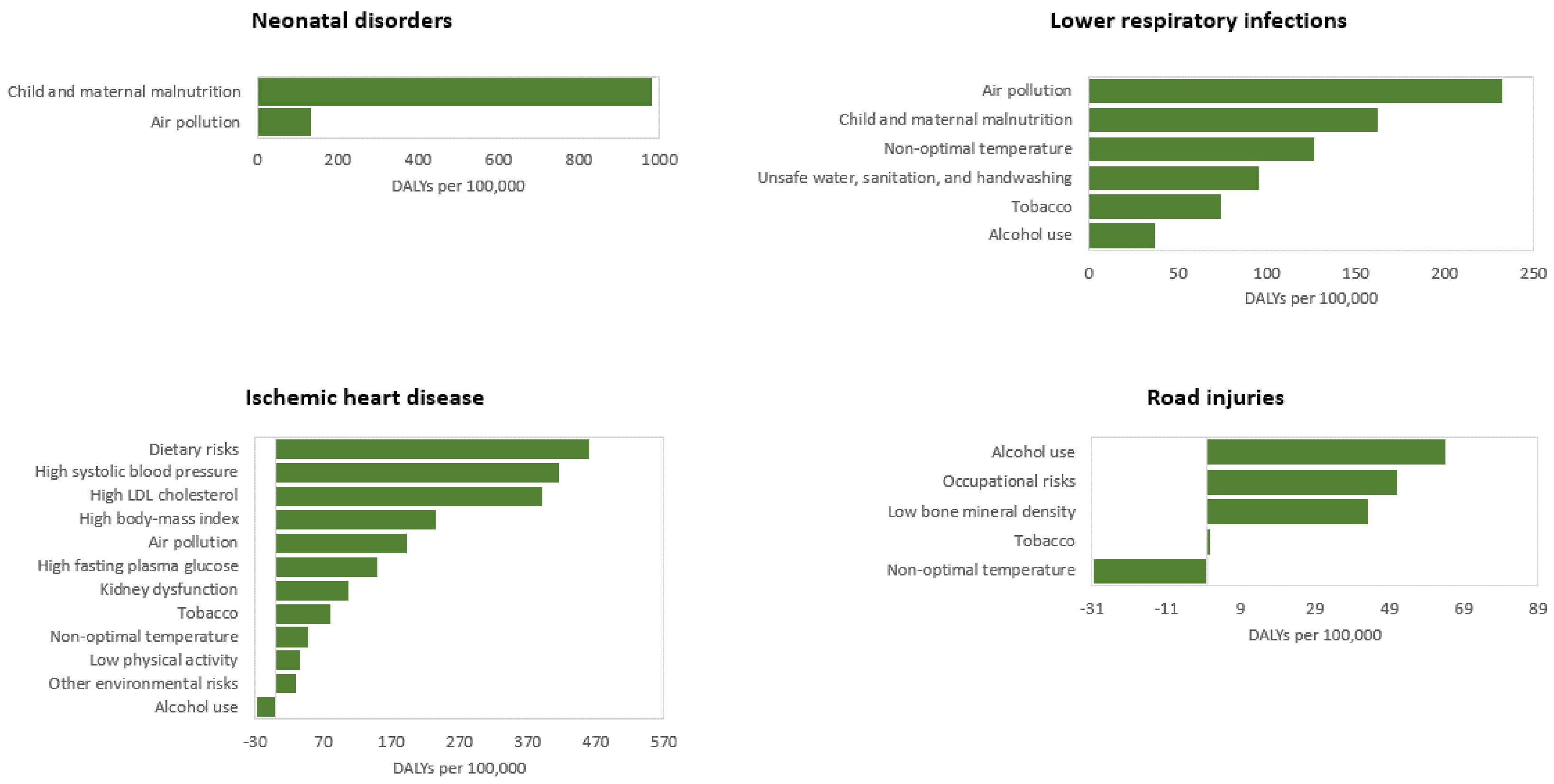

Supplement: Supplementary file 1 [file Image_1.pdf]
