# Supplementary material for: Estimating mortality and disability in Peru before the COVID-19 pandemic: a systematic analysis from the Global Burden of the Disease Study 2019
Source: Front Public Health. 2023 Jun 22;11:1189861. doi: 10.3389/fpubh.2023.1189861 (PMC10325574; doi:10.3389/fpubh.2023.1189861)

# Supplementary figures

**Supplementary Figure** **1**. Population pyramid from Peru in 1990 and 2019

**Supplementary Figure 2**. Top 15th leading causes of prevalent cases in Peru (1990 and 2019), with change (%) in the number of prevalent cases and all-ages prevalence.


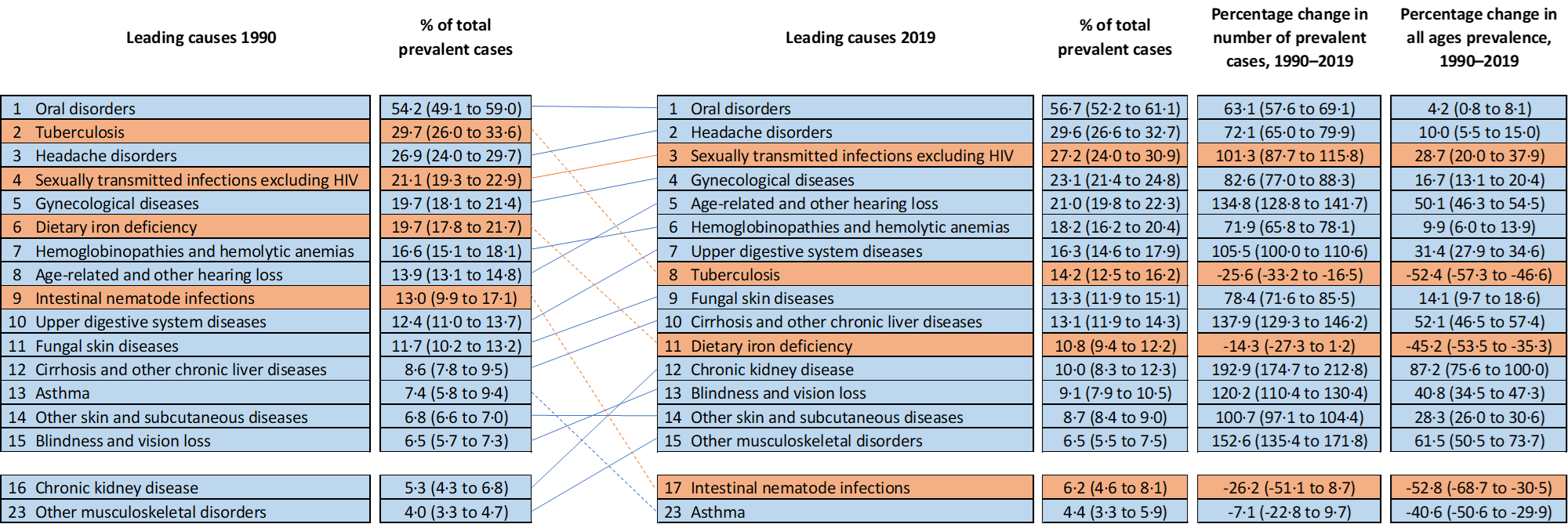

The level-3 causes of GBD disaggregation are presented. Lines between time periods connect causes; solid lines represent increases in rank, and dashed lines represent decreases.

**Supplementary Figure 3**. Top 15th leading causes of incident cases in Peru (1990 and 2019), with change (%) in the number of incident cases and all ages-incidences


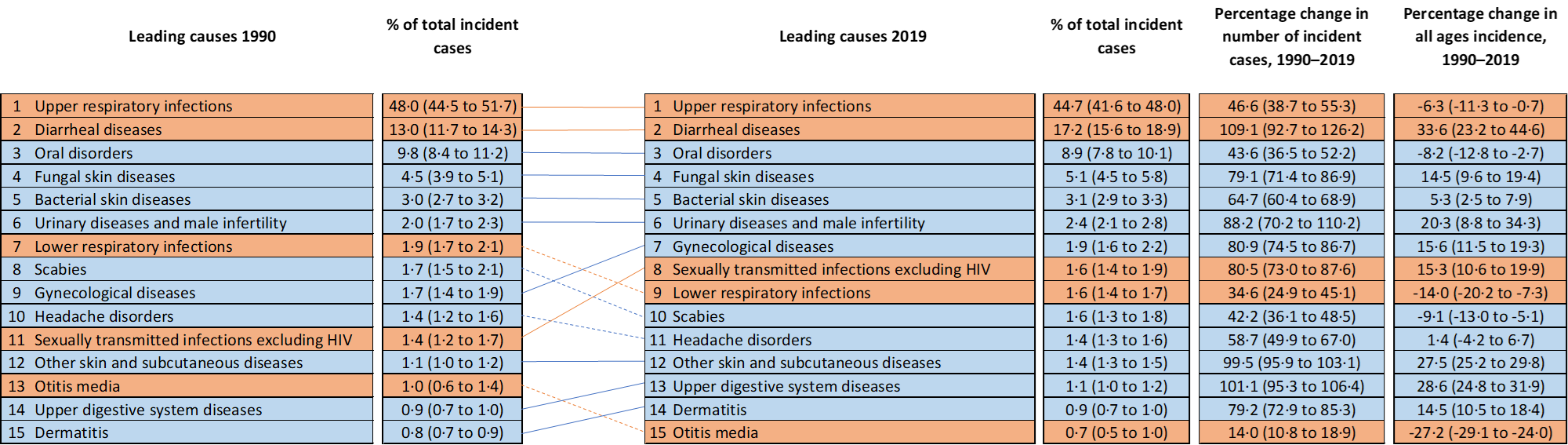

The level-3 causes of GBD disaggregation are presented. Lines between time periods connect causes; solid lines represent increases in rank, and dashed lines represent decreases.

**Supplementary Figure 4**. Percentage change in the absolute number of risk-attributable DALYs in Peru from 1990-2019 due to population growth, population aging, changes in exposure to each GBD risk factor, and changes in risk-deleted DALY rates for both sexes and all causes combined.


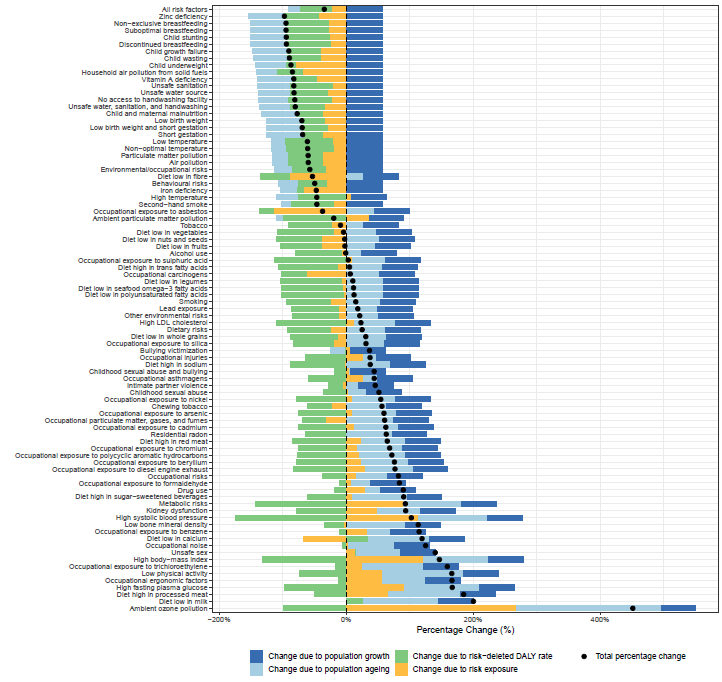


**Supplementary Figure 5**. Age-standardized rate of top four causes of DALYs attributable to risk factors in Peru in 1990 and 2019.


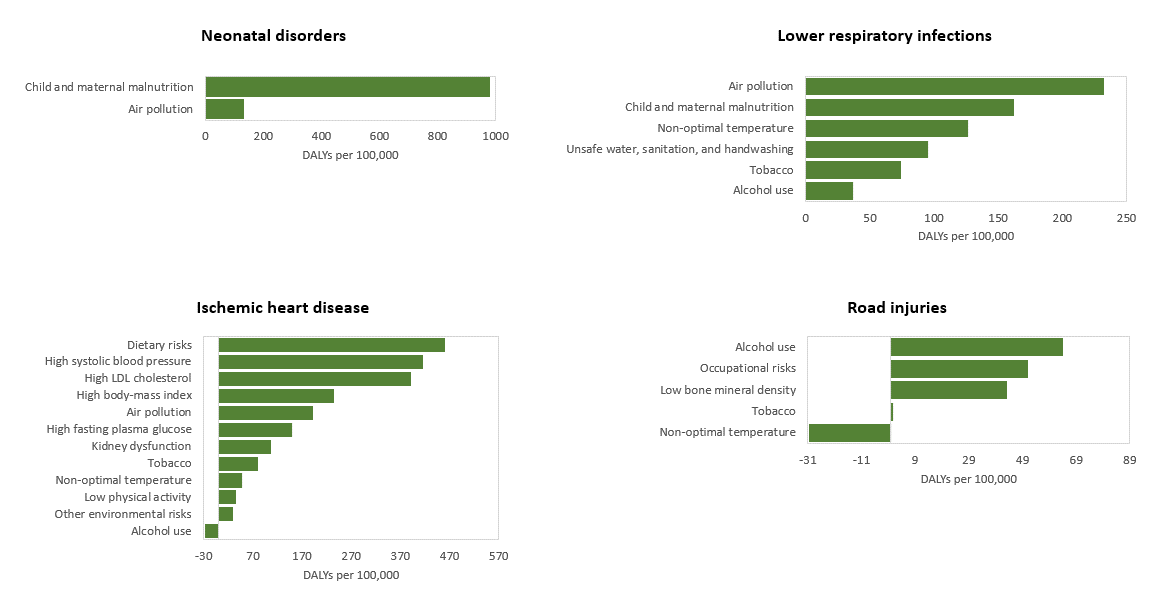

Supplement: Supplementary file 2 [file Table_1.docx]
